# The *Hedyotis diffusa* Willd. (Rubiaceae): A Review on Phytochemistry, Pharmacology, Quality Control and Pharmacokinetics

**DOI:** 10.3390/molecules21060710

**Published:** 2016-05-30

**Authors:** Rui Chen, Jingyu He, Xueli Tong, Lan Tang, Menghua Liu

**Affiliations:** 1 State Key Laboratory of Organ Failure Research, Guangdong Provincial Institute of Nephrology, Southern Medical University, Guangzhou 510515, Guangdong, China; ruichen1127@126.com (R.C.); xueli_tong@126.com (X.T.); tl405@smu.edu.cn (L.T.); 2 Guangdong Provincial Key Laboratory of New Drug Screening, School of Pharmaceutical Sciences, Southern Medical University, Guangzhou 510515, Guangdong, China; 3 Bioengineering Research Centre, Guangzhou Institute of Advanced Technology, Chinese Academy of Sciences, Guangzhou 511458, Guangdong, China; jy.he@giat.ac.cn

**Keywords:** *H. diffusa*, phytochemistry, pharmacology, quality control, pharmacokinetics

## Abstract

*Hedyotis diffusa* Willd (*H. diffusa*) is a well-known Chinese medicine with a variety of activities, especially its anti-cancer effect in the clinic. Up to now, 171 compounds have been reported from *H. diffusa*, including 32 iridoids, 26 flavonoids, 24 anthraquinones, 26 phenolics and their derivatives, 50 volatile oils and 13 miscellaneous compounds. *In vitro* and *in vivo* studies show these phytochemicals and plant extracts to exhibit a range of pharmacological activities of anti-cancer, antioxidant, anti-inflammatory, anti-fibroblast, immunomodulatory and neuroprotective effects. Although a series of methods have been established for the quality control of *H. diffusa*, a feasible and reliable approach is still needed in consideration of its botanical origin, collecting time and bioactive effects. Meanwhile, more pharmacokinetics researches are needed to illustrate the characteristics of *H. diffusa in vivo*. The present review aims to provide up-to-date and comprehensive information on the phytochemistry, pharmacology, quality control and pharmacokinetic characteristics of *H. diffusa* for its clinical use and further development.

## 1. Introduction

*Hedyotis diffusa* Willd (*H. diffusa*, Family Rubiaceae), known as *Oldenlandia diffusa* (Willd) Roxb, is a well-known Chinese medicine used for the treatment of inflammation-linked diseases, such as hepatitis, appendicitis and urethritis, for thousands of years in China [1]. In our previous studies, the water extract of *H. diffusa* has been proved to have an obvious protective effect in lipopolysaccharide-induced renal inflammation in mice. Recently, *H. diffusa* has gained increasing attention for its properties of anti-proliferative activity in cancer cells and anti-tumor activity in tumor-bearing animals [2,3,4,5]. It has been proved as the most commonly prescribed single Chinese herb used for colon cancer and breast cancer patients [6,7], according to the statistics from the National Health Insurance Research Database of Taiwan.

*H. diffusa* is an annual herb, widely distributed in the orient and tropical Asia, such as China, Japan and Indonesia [1,8]. Generally, the plant grows in humid fields and ridges of farmlands, ascending to procumbent, to 50 cm tall; the stem is slightly flattened to terete, glabrescent to glabrous and the papilla was observed in the transverse section of the stem; the leaves are opposite, sessile or subsessile and blade drying membranous, linear, narrowly elliptic, 1–4 × 0.1–0.4 cm; the flowers with pedicels are pairs in axillary racemes and the corolla is white [1,9]. Together with these phenotypic characteristics of *H. diffusa*, methods of thin-layer chromatography (TLC) [10], gas chromatography-mass spectrometer (GC-MS) [11], high performance liquid chromatography (HPLC) [9] and DNA sequencing [8,12] have been developed to differentiate *H. diffusa* from related species (e.g., *Hedyotis corymbosa* (L.) Lam) to give the right prescription for illnesses.

Although there are numbers of published scientific literature on the chemical constituents, pharmacological activities and quantitative analysis of *H. diffusa*, a systematic and updated review is unavailable. Therefore, the aim of this review is to extensively summarize the phytochemistry, pharmacology, quality control and pharmacokinetic characteristics of *H. diffusa*, as well as being an evidence for clinical uses and further researches of this herb.

## 2. Phytochemistry

With the advancement of analysis technologies like mass spectrometer (MS), liquid chromatograph–mass spectrometer (LC-MS), nuclear magnetic resonance–mass spectrometer (NMR-MS) *etc.*, many studies on *H. diffusa* revealed numbers of important phytochemicals, including iridoids, triterpenes, flavonoids, anthraquinones, phenolic acids and their derivatives, sterols, alkaloids, volatile oils, polysaccharides, cyclotides, coumarins and alkaloids. The detailed information for these compounds is summarized in Table 1.

### 2.1. Iridoids and Triterpenes

**Figure 1:**
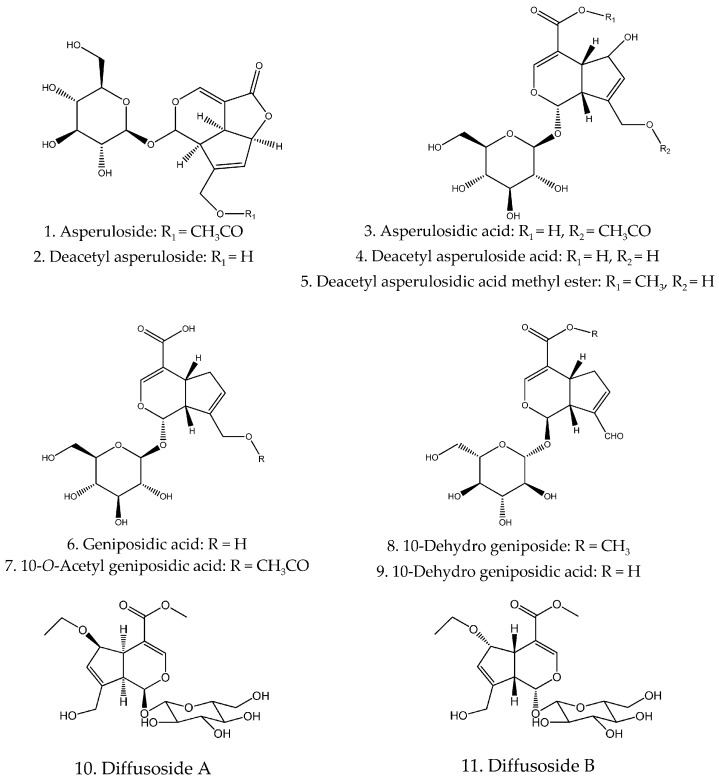

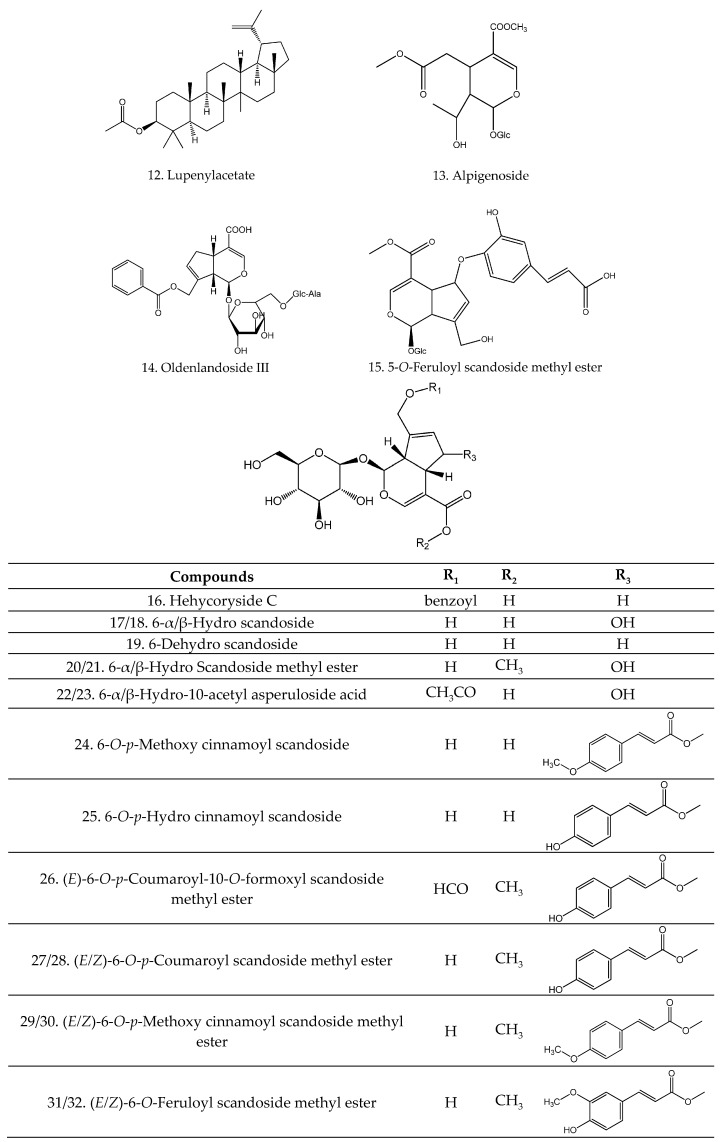

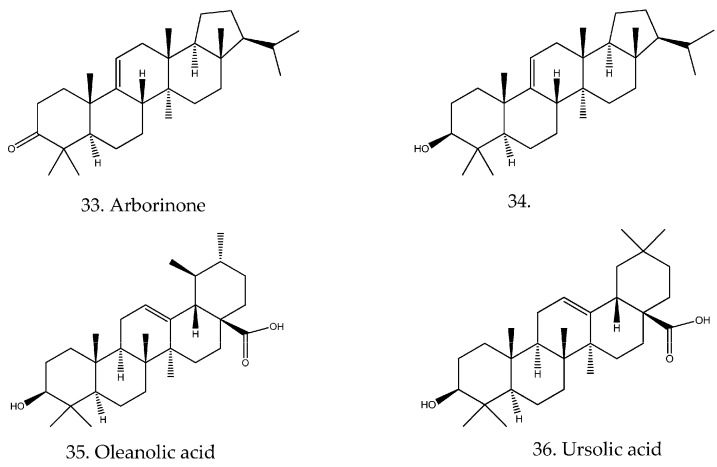
Chemical structures of iridoids and triterpenes in *H. diffusa*.

Iridoids are one of the most important components in *H. diffusa* with various bioactivities, such as anti-oxidant, neuroprotective and anti-inflammatory effects [33,53]. Accompanied with the analysis of the NMR spectra of the pure compounds, the methods of tandem mass spectrometry (MS^n^) and time-of-flight mass spectrometry (TOF/MS) have become more popular for the identification of these compounds [11,14,15,25,52]. To date, thirty-two iridoids and their iridoid glucosides (**1**–**32**) have been isolated and identified from *H. diffusa* (Figure 1).

Four triterpenes, named arborinone (**33**), isoarborinol (**34**), oleanolic acid (**35**) and ursolic acid (**36**), were isolated from *H. diffusa* and their structures were established by 1D-, 2D-NMR spectroscopic analysis and high-resolution electrospray ionization mass spectroscopy (HRESIMS) [53].

### 2.2. Flavonoids

**Figure 2:**
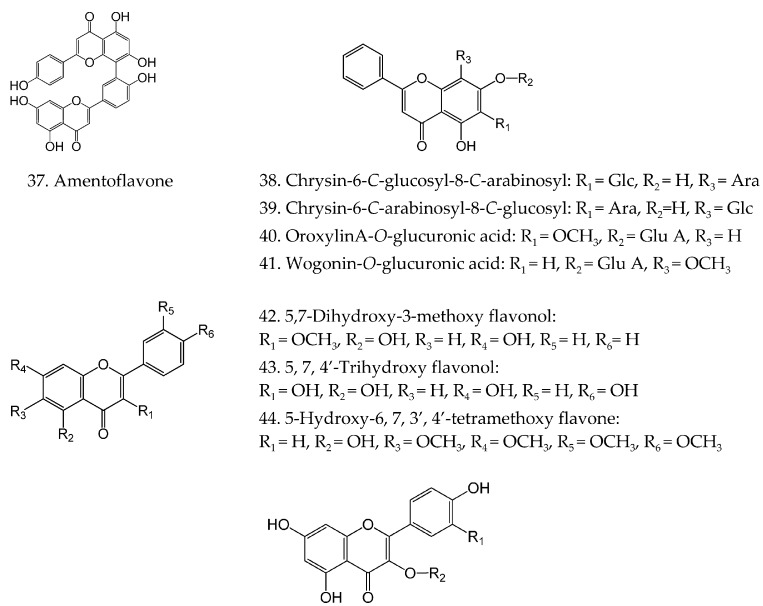
Chemical structures of flavonoids in *H. diffusa*.

Flavonoids are a major group presented in *H. Diffusa*, and most of them are derivatives of the flavonol aglycones of kaempferol and quercentin. Recently, other aglycones, such as chrysin, oroxylin and wogonin, have been characterized by ultra-performance liquid chromatography–diode array detector/quadrupole time-of-flight mass spectrometry (UPLC-DAD/Q-TOF-MS). To date, twenty-six flavonoids (**37–62**) with various substitutions have been identified and their chemical structures are prescribed in Figure 2.

### 2.3. Anthraquinones

**Figure 3:**
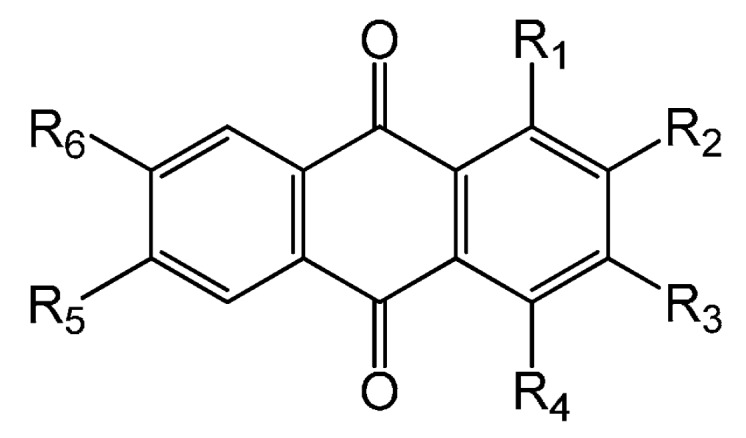
Chemical structures of anthraquinones in *H. diffusa*.

Anthraquinones are also a major group of bioactive components in *H. diffusa*. Up to now, twenty-four anthraquinones with various substitutions (**63–86**) have been obtained and identified from this herb. These compounds have a typical characteristic of the 9, 10-anthraquinone skeleton with the presence of hydroxy, methyl and/or methoxy groups, for example, 2-hydroxy-3-methoxy-6-methyl anthraquinone (**77**). Their chemical structures are shown in Figure 3.

### 2.4. Phenolic Acids and Their Derivatives

**Figure 4:**
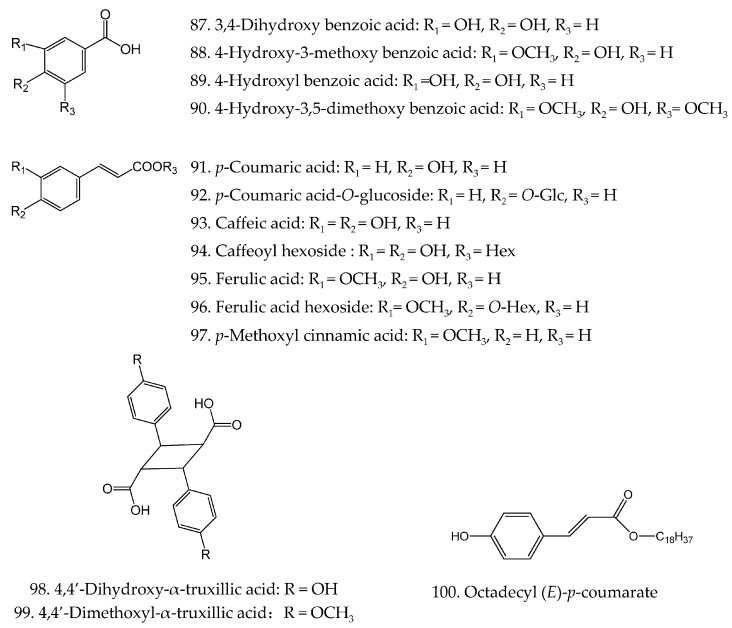

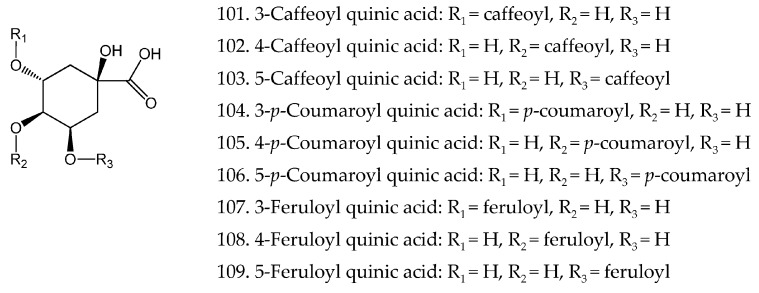
Chemical structures of phenolic acids and their derivatives in *H. diffusa*.

Phenolic acids are very common and important secondary metabolites in nature. To date, twenty-three phenolic acids (**87**–**109**) have been identified from the herb of *H. Diffusa*, including four benzoic acid derivatives (**87**–**90**), coumaric acid (**91**) and its derivative (**92**), caffeic acid (**93**) and its derivative (**94**), ferulic acid (**95**) and its derivative (**96**), *p*-methoxyl cinnamic acid (**97**), two truxillic acid derivatives (**98**–**99**), octadecyl (*E*)-*p*-coumarate (**100**) and nine quinic acid derivatives (**101**–**109**). Their chemical structures are prescribed in Figure 4.

### 2.5. Polysaccharides

The polysaccharides in *H. diffusa* have been researched for their immuno-enhancing activity. They are mainly composed of glucose, galactose and mannose, with the content of 15.10% determined by the spectrophotometry method at 490 nm [54]. Up to now, only one homogeneous polysaccharide, ODP-1, has been separated from *H. diffusa*, with the relative molecular weight of 20.88 kDa. It consists of mannose, rhamnose, galacturonic acid, glucose, galactose and arabinose, with the molar ratio of 0.005:0.033:0.575:1.000:0.144:0.143 [50].

### 2.6. Essential Oils

The reports of essential oils in this plant were mainly on isolating many fatty acids, fatty acid esters, *etc.* [11]. Yang *et al.* [49] identified 29 compounds representing 81.45% of the total oil content by GC/MS combined with the Kovats Retention index. *n*-Hexadecanoic acid (**119**) (31.22%), oleic acid (157) (6.74%), tetracosane (**161**) (4.94%) and 9,12-octadacadienoic acid (**125**) (4.87%) were found to be the main constituents. Liu *et al.* [48] compared the constituents and their content in *H. diffusa* collected from the provinces of Guangdong, Jiangxi and Guangxi in China and also revealed that the oil of *H. diffusa* was mainly composed of fatty acids with an oil extraction rate from 0.25% to 0.30%.

### 2.7. Cyclotides

Three novel cyclotides from *H. diffusa*, named CD1 (**165**), CD2 (**166**) and CD3 (**167**), with an anti-cancer effect on prostate cancer cells, were reported by Hu *et al*. [51]. The primary sequences were GAFLKCGESCVYLPCLTTVVGCSCQNSVCYRD, GAVPCGETCVYLPCITPDIGCSCQNKVCYRD and G-TSCGETCVLLPCLSSVLGCTCQNKRCYKD for DC1, DC2 and DC3, respectively.

### 2.8. Miscellaneous

**Figure 5:**
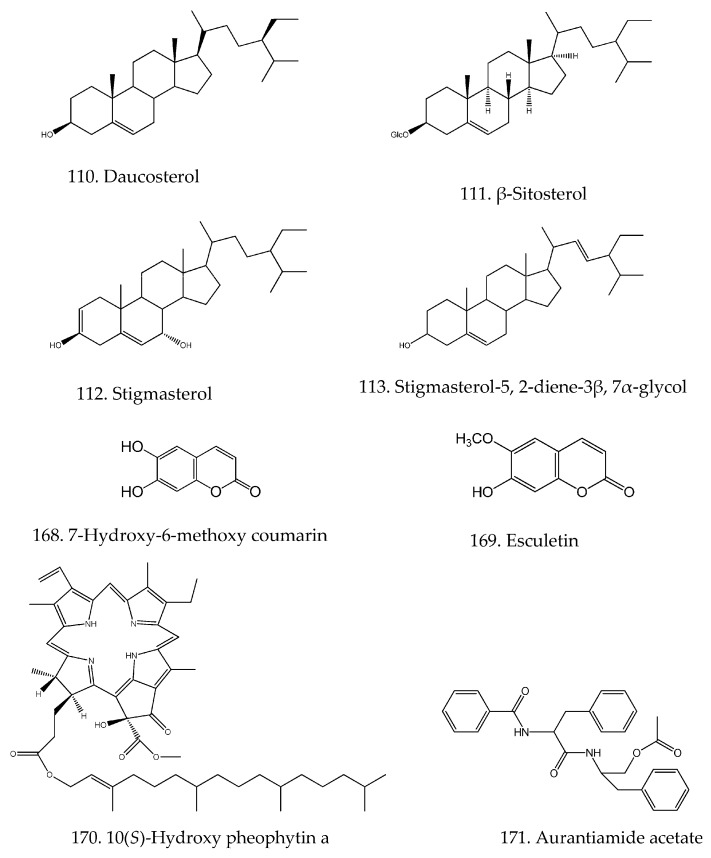
Chemical structures of miscellaneous components in *H*. *diffusa*.

Only four sterols of daucosterol (**110**), β-sitosterol (**111**), stigmasterol (**112**) and stigmasterol-5,2-diene-3β, 7α-glycol (**113**), two coumarins of 7-hydroxy-6-methoxy-coumarin (**168**) and esculetin (**169**) and two alkaloids of 10-hydroxypheophytin a (**170**) and aurantiamide acetate (**171**) have been purified and characterized from *H. diffusa* and their structures are shown in Figure 5.

## 3. Pharmacology

*H. diffusa* has long been used therapeutically in China, due to its broad spectrum of biological and pharmacological activities. Now we have enlisted an overview of the modern pharmacological studies in the following sections (Table 2).

### 3.1. Anti-Cancer Activity

#### 3.1.1. Anti-Colorectal Cancer Activity

*H. diffusa* has been used as a major formula for the clinical treatment of colorectal cancer (CRC). *In vitro*, ethanol extract of *H. diffusa* treatment significantly suppresses proliferation and induced apoptosis of HT-29 cells, resulting in DNA fragmentation, loss of plasma membrane asymmetry, collapse of mitochondrial membrane potential, activation of caspase-9 and caspase-3, increase of the ratio of pro-apoptotic Bax to anti-apoptotic Bcl-2, reduction of the mRNA expression levels of cyclin D1, cyclin-dependent kinase 4 and B-cell lymphoma-2 (Bcl-2), upregulation of the expression levels of Bcl-2-associated X protein, prevention of G1–S progression, and reduction of mRNA expression of pro-proliferative PCNA, Cyclin D1 and CDK4. These results indicated that the anti-colorectal cancer cells effect of *H. diffusa* might be carried out via multiple approaches, such as the mitochondria-dependent pathway, IL-6/STAT3 pathway and cell cycle arrest [2,55,56,57,58]. The mechanism was also confirmed by animal experiments [58,59]. Meanwhile, the ethanolic extract of *H. diffusa* displayed an inhibition effect on CT-26 cells with inhibitory rates from 35.46% ± 3.59% to 71.84% ± 3.12% at different concentrations (0.06 mg/mL, 0.08 mg/mL, 0.10 mg/mL, 0.12 mg/mL, 0.14 mg/mL, 0.16 mg/mL, 0.18 mg/mL and 0.20 mg/mL) and showed a stronger inhibition effect with an increase of concentration [60]. Li *et al*. [61] revealed that the ethanolic extract treatment could overcome 5-fluorouracil resistance in HCT-8/5-FU cells by downregulating the expression of P-gp and ABCG2. In addition, 2-hydroxymethy-1-hydroxy anthraquinone (IC_50_ 45 μM) and ursolic acid (IC_50_ 71 μM)) isolated from *H. diffusa* exhibited inhibition effects on Caco-2 cell proliferation [5], and the mechanism of the inhibition effect for ursolic acid might include the cleavage of the Poly (ADP-ribose) Polymerase (PARP) [62]. 

#### 3.1.2. Anti-Leukemia Activity

The anti-leukemia effects of both aqueous and ethanolic extracts of *H. diffusa* have been investigated in several cancer cell lines. *H. diffusa* aqueous extract treatment with 0.01–4150 μg/mL restrained the growth of the CEM cells by enhancing the expression of P53 *in vitro* [63] and influenced murine leukemia WEHI-3 cells, as well as promoting T- and B-cell proliferation in leukemic mice administrated with 16 and 32 mg/kg *in vivo* [64]. The ethanolic extract of *H. diffusa* could trigger an arrest of HL-60 cells at the G_0_/G_1_ phase and sub-G_1_ population, provoke DNA condensation and DNA damage, but elevate the activities of caspase-3, caspase-8 and caspase-9, thus, inhibiting the cell proliferation of HL-60 cells with the half maximal inhibitory concentration (IC_50_) value of 4.62 mg/mL [65,66]. Wang *et al.* [67] found that 2-hydroxy-3-methyl anthraquinone treatment (0–80 μM) could enhance apoptosis of U937 cells in a dose-dependent manner through the activation of p-p38MAPK and downregulation of p-ERK1/2. Further study verified it could alter the expression of Fas/FasL and activation of caspase-8, thus inducing THP-1 cell apoptosis [68].

#### 3.1.3. Anti-Liver Cancer Activity

Li *et al.* [69] reported the inhibition of aqueous extract of *H. diffusa* on blood metastasis in H22 mice. The body and immune organs weights increased after administration with *H. diffusa* extract at three doses of 0.25, 0.5 and 1.0 mg/kg. *In vitro*, the aqueous extract of *H. diffusa* treatment (1.25–10 mg/mL) remarkably inhibited HepG2 cell proliferation in a dose-dependent manner, probably via the arrest of HepG2 cells at the G_0_/G_1_ phase and the induction of S phase delay [70]. Treatment with total flavones extract from *H. diffusa* could reverse the invasion of MHCC97-H cells in epithelial-mesenchymal transition induced by TGF-β1 at the dose of 200μg/mL, and the effect might be carried out by decreasing the level of E-cadherin protein and increasing the expression of vimentin protein [71]. Li *et al.* found that both 1,3-Dihydroxy-2-Methylanthraquinone (79 and 157 μmol/L) and ethyl acetate extract (100 and 200 μg/mL) induced apoptosis on HepG2 cells, resulting in upregulation of Bax, p53, Fas, FasL, p21 and cytoplasmic cytochrome C levels and caspase-3, -8, -9 proteases activities, while downregulation of Bcl-2, mitochondrial cytochrome C, cyclin E and CDK 2 in a dose-dependent manner [72]. Nine compounds from *H. diffusa*, namely, ethyl 13 (*S*)-hydroxy-chlorophyllide a, 2-methyl-3-methoxy anthraquinone, 2-hydroxymethyl anthraquinone, 2-hydroxy-3-methyl anthraquinone, 2-hydroxymethy-1-hydroxy anthraquinone, 2-hydroxy-1-methoxy anthraquinone, 2-hydroxy-3-methyl-1-methoxy anthraquinone, oleanolic acid and ursolic acid, have been researched for their anti-liver cancer effect within the concentration range from 1 to 200 μM. As a result, ursolic acid exhibited a strong inhibition of HepG2 cell survival (IC_50_ 36.63 μM) [5]. Another study revealed that the inhibitory activity of 2-hydroxy-3-methyl anthraquinone (IC_50_ 51 μM) and 2-hydroxy-1-methoxy anthraquinone (IC_50_ 62 μM) might be achieved by activity against protein tyrosine kinases v-src and pp60src [38].

#### 3.1.4. Anti-Lung Cancer Activity

Aqueous extract of *H. diffusa* treatment (0–200 μg/mL) showed a suppression effect on A549 and H1355 cells in a concentration-dependent manner. But this effect was not found in LLC cells [66]. Further, Shi *et al.* [38] confirmed that two compounds of 2-hydroxy-3-methyl anthraquinone (IC_50_ 66 μM) and 2-hydroxy-1-methoxy anthraquinone (IC_50_ 79 μM) from *H. diffusa* could induce apoptosis on SPC-1-A cells with a close relationship to the mitochondrial apoptotic pathway.

#### 3.1.5. Anti-Breast Cancer Activity

Anthraquinones, iridoid glucosides, stigmasterols and alkaloid/flavonoid extracts were evaluated for anti-breast cancer using human breast cancer cell line MCF7. Dong *et al.* [73] found that the crude alkaloid/flavonoid extract, but not its three major components, possessed antitumor activity against the human breast cancer cell line MCF7. However, Liu *et al.* [74] observed that methyl anthraquinone from *H. diffusa* exhibited an inhibition effect on MCF7 cells with half maximal effective concentration (EC_50_) of 18.62 ± 2.71 and 42.19 ± 3.84 μM for 24 and 48 h, respectively, and induced MCF-7 cells apoptosis via the Ca^2+^/calpain/caspase-4 pathway. Moreover, compounds of 2-hydroxy-3-methyl anthraquinon (IC_50_ 57 μM) and 2-hydroxy-1-methoxy anthraquinone) (IC_50_ 65 μM) inhibited protein tyrosine kinases v-src and pp60src and the growth of Bcap37 cells [38].

#### 3.1.6. Anti-Cervical Tumor Activity

Zhang *et al.* [3] discovered that the aqueous extract of *H. diffusa* treated (0.5 g/kg bw) by intragastric administration on human cervical carcinoma xenograft in nude mice showed an inhibitory effect on cervical cancer cells and induced apoptosis of Hela cells. Meanwhile, anthraquinones, especially 2-hydroxymethy-1-hydroxy anthraquinone, showed a strong inhibitory effect on Hela cells with IC_50_ 45 μM *in vitro* [5].

#### 3.1.7. Anti-Prostate Cancer Activity

The potential anti-prostate cancer effect of *H. diffusa*, mainly the active compounds, has previously been provided on a variety of cell lines. 2-Methyl-3-methoxy anthraquinone (IC_50_ 64.72–105.90 μM), 2-hydroxy-3-methyl anthraquinone (IC_50_ 28.82–159.20 μM) and ursolic acid (IC_50_ 22.33–36.08 μM) exhibited inhibitory effects on DU145, PC-3 and LNCaP cells [5]. 6-*O*-(*E*)-*p*-coumaroyl scandoside methyl esterand 10(*S*)-hydroxy pheophytin a showed an anti-proliferation effect on PC-3 cells in a dose-dependent manner from 0 to 60 μM, while 10(*S*)-hydroxy pheophytin a also showed a strong anti-proliferation effect on LNCaP cells with a significant effect, with an IC_50_ value of 20 μM [52]. Hu *et al.* [51] isolated three cyclotides (DC 1-3) and studied their anti-prostate cancer effect. Thus, three cyclotides, especially DC 3 (1 mg/kg) showed inhibition against PC3, DU145 and LNCap cells. In addition, DC3 significantly inhibited development of the tumor in weight and size in the model of a prostate xenograft, and showed significant anti-cancer effect (*p* < 0.01) at a dose of 1 mg/kg, with 40.23% inhibition of the rate of tumor growth (weight).

#### 3.1.8. Anti-Multiple Myeloma Activity

Up to now, the anti-multiple myeloma effect of *H. diffusa* has been proved in RPMI 8226 cells. The polysaccharides extracts (1, 2 and 3 mg/mL) [75], the compound of 2-hydroxymethyl-1-hydroxy anthraquinone (1–200 μM) [5], as well as *H. diffusa* injection (20, 40 and 60 μL/mL) [76], exhibited an inhibitory effect on RPMI 8226 cells growth in a dose-dependent manner.

#### 3.1.9. Other Anti-Cancer Effects

Other anti-cancer effects have also been reported during these years. The ethanolic extract of *H. diffusa* (0–200 μg/mL) suppressed the proliferation of B16F10 cells in a dose-dependent manner [66]. The lipophilic extract (50 and 100 mg/kg) and crude polysaccharide (31.25 and 62.5 mg/kg) from *H. diffusa* showed anti-tumor activities on S-180 cells and a protective effect on chemotherapeutic damage [77]. *H. diffusa* injection could induce the apoptosis of MG-63 cells by increasing the Bax gene expression in a concentration-dependent manner from 50 to 400 μg/mL [78,79]. When it is used with cisplatin, the combined use exhibited a stronger inhibitory effect than the single agents. This might be due to its property of elevating the levels of Bax, Bad, caspase-3 and caspase-8 expression and decreasing the levels of Bcl-xl and Bcl-2 [80]. Meanwhile, Zhang *et al.* [4] found that the aqueous extract of *H. diffusa* (2–8 mg/mL) inhibited the growth of U87 cells in a dose-dependent manner by inducing mitochondrial apoptosis via the AKT/ERK pathways. Moreover, the compound, 4-vinyl phenol, was demonstrated to have anti-angiogenic activity in human endothelial cells of HUVEC (IC_50_ 15.31 μg/mL) and HMEC_-1_ (IC_50_ 21.43 μg/mL), breast tumor-bearing BALB/c mice (0.2–2 mg/kg), C57BL/6 mice (20–100 μg/mL matrigel) and zebrafish embryo models (6.25–12.5 μg/mL matrigel), and this effect had a close relationship with the PI3K/AKT pathway [81].

### 3.2. Immunomodulatory Effect

Lin *et al.* [64] found that aqueous extract of *H. diffusa* (16 and 32 mg/kg) affected immune responses by promoting T- and B-cell proliferation in leukemic mice in WEHI-3-generated leukemia mice. Meanwhile, Kuo *et al.* [82] discovered that the ethanolic extract (16, 32 and 64 mg/kg) could also promote immune responses in normal BALB/c mice by promoting CD11b, CD19 and Mac-3 levels, increasing phagocytosis activity of macrophages obtained from the peritoneal cavity and increasing NK cell activity and B- and T-cell proliferation. The polysaccharides extracts (2.25, 4.5 and 9.0 mg/mL) could improve the clearance index, phagocytic index and the index of the thymus and spleen of immunosuppression mice [50]. When inmmunosuppressed mice were orally administrated total flavonoids of *H. diffusa* (15, 30 and 60 mg/kg), the levels of interleukin-2 (IL-2) and interferon-γ (INF-γ) were enhanced and the proliferation of T and B lymphocytes was increased, indicating the immunomodulatory effect of total flavonoids [83].

### 3.3. Antioxidant Effect

The aqueous, methanolic and 80% acetonic extracts were evaluated for antioxidant activity and the extraction from 80% alcohol (0.1–4.5 mg/mL) showed the strongest antioxidant activity, by 2,2-diphenyl-1-picrylhydrazyl (DPPH) assay [84]. Yu *et al.* [85] compared the antioxidant effects of aqueous, alcoholic, acetonic, chloroform, ether and petroleum benzene extracts from *H. diffusa*, and found that the acetonic extracts (0.03%–0.18%), especially the 0.12% acetone extract, had the strongest antioxidant effect, by determination of peroxide value. In addition, the aqueous extract (0.3–10 mg/mL) treatment could protect human hepatocyte cells from H_2_O_2_-induced cytotoxicity in a dose-dependent manner as the probable result of the improvement activity of the aqueous extract of *H. diffusa* on the antioxidant defense system by reversing H_2_O_2_-induced activation of the MEK/ERK pathway and H_2_O_2_-induced inhibition of the P13-K/AKT/GSK3β pathway in LO_2_ cells [86]. The antioxidant effect of *H. diffusa* may be due to its compounds, like flavonoids and iridoids. Three flavonol glycosides (quercetin 3-*O*-sambubioside, kaempferol-3-*O*-[2-*O*-(E-6-*O*-feruloyl)-β-d- glucopyranosyl]-β-d-galactopyranoside and quercetin 3-*O*-sophoroside) and six known iridoid glycosides (asperuloside, asperulosidic acid methyl ester, (*E*)-6-*O*-*p*-methoxy cinnamoyl scandoside methyl ester, (*E*)-6-*O*-feruloyl scandoside methyl ester and (*E*)-6-*O*-coumaroyl scandoside methyl ester) were determined for their antioxidant effects on xanthine oxidase inhibition, xanthine-xanthine oxidase cytochrome c and TBA-MDA systems. In consequence, asperuloside (IC_50_ 118.5 ± 0.70 μM) and kaempferol-3-*O*-(2-*O*-β-d-glucopyranosyl)-β-d-galactopyranoside (IC_50_ 98.7 ± 0.16 μM) showed a minor anti-lipid peroxidation effect and quercetin di-glycosides exerted a remarkable antioxidant effect as superoxide anion scavengers [33].

### 3.4. Anti-Inflammatory Effect

The aqueous extract (5.0 g/kg bw) treatment exhibited an anti-inflammatory effect in lipopolysaccharide-induced renal inflammation of mice by significantly suppressing the production of tumor necrosis factor-α (TNF-α), IL-1, IL-6 and monocyte chemotactic protein 1 (MCP-1) in renal tissues, as well as significantly promoting the production of IL-10 in serum and renal tissues. Moreover, two main chemotypes, including eight flavonoids and four iridoid glycosides were found in renal tissues after *H. diffusa* treatment, indicating that the anti-inflammatory effect may be due to these constituents [87]. *In vitro,* Chen *et al.* found that the flavonoids extract treatment (50−100 μg/mL) on LPS-stimulated RAW 264.7 cells reduced expression of iNOS, TNF-α, IL-6 and IL-1β, as well as suppressing phosphorylation of IκB p38, JNK and ERK1/2 in a concentration-dependent manner, indicating that the anti-inflammatory activity of total flavonoids had a close relationship with the NF-κB- and MAPK-signaling pathways [88].

### 3.5. Others

Five flavonol glycosides (kaempferol-3-*O*-[2-*O*-(6-*O*-*E*-feruloyl)-β-d-glucopyranosyl]-β-d-galactopyranoside, quercetin-3-*O*-[2-*O*-(6-*O*-*E*-feruloyl)-β-d-glucopyranosyl]-β-d-galactopyranoside, quercetin-3-*O*-[2-*O*-(6-*O*-*E*-feruloyl)-β-d-glucopyranosyl]-β-d-glucopyranoside, kaempferol-3-*O*-(2-*O*-β-d-glucopyranosyl)-β-d-galactopyranoside and quercetin-3-*O*-(2-*O*-β-d-glucopyranosyl)-β-d-galactopyranoside) and four *O*-acylated iridoid glycosides (6-*O*-Z-*p*-methoxy cinnamoyl scandoside methyl ester, 6-*O*-*E*-*p*-methoxy cinnamoyl scandoside methyl ester, 6-*O*-*Z*-*p*-coumaroyl scandoside methyl ester and 6-*O*-*E*-*p*-coumaroyl scandoside methyl ester) isolated from *H. diffusa* exhibited a significant neuroprotective effect on l-glutamate-damaged rat cortical cells in the concentration from 0.1 to 10 μM; further, the structure–activity study proved di-OH in the B ring and an acyl substituent in flavonoids, a *p*-methoxy group in the aromatic ring and a trans double bond in the acyl moiety of acylated iridoid glycosides might be crucial for the biological response [34]. Wu *et al.* [89] found the inhibitory effect of oleanolic acid (2 and 8 μg/mL) isolated from *H. diffusa* against ras-transformed fibroblasts on R6 cells, and this inhibition might cause normal cells to secrete an inhibitory factor against the transformed cells, but did not require direct cell–cell contact.

## 4. Quality Control

Quality control of herbal medicines is necessary to ensure their stability, efficiency and safety. Modern analytical techniques provide simpler, more accurate and reliable methods for the quality control for *H. diffusa*. Besides the macroscopic and microscopic characters of *H. diffusa* [9], DNA sequence has become a powerful tool for the distinguishing *H. diffusa* from counterfeits, such as *H. corymbosa* and *H. tenelliflora* [8,12]. Chemical fingerprint is a comprehensive method accepted by the Food and Drug Administration, European Medicines Agency, and China Food and Drug Administration [90]. It can provide information about the types of compounds, as well as their relative ratios. A HPLC-MS fingerprint method was applied to 10 batches of *H. diffusa* materials from nine regions in China. The results showed that this method could differentiate samples from different geographical origins or processing methods [91]. Liang *et al.* [92] analyzed the chemical fingerprints of 17 batches of *H. diffusa* and found that the contents of asperuloside and (*E*)-6-*O*-*p*-coumaroyl scandoside methyl ester were quite different in samples collected from different habitats. 

The quantitative analysis for the quality control of *H. diffusa* has mostly focused on the diversity of components by a series of analytical methods, such as UV, HPLC, TLC and LC/MS. Up to now, triterpenes (ursolic acidand oleanulic acid), iridoids ((*E*)-6-*O*-*p*-coumaroyl scandoside methyl ester), geniposidic acid, deacetyl asperulosidic acid methyl ester, asperuloside acid, asperulosideand (*E*)-6-*O*-feruloyl scandoside methyl ester), phenolic acid (*p*-coumaric acid and ferulic acid), flavonoids (quercetin, rutin, quercetin-3-*O*-β-d-glucopyranside, quercetin-3-*O*-sambubioside, kaempferol, kaempferol-3-*O*-β-d-glucopyranside), anthraquinones (2-hydroxy-3-methoxy-7-methyl anthraquinone and 2-hydroxy-1-methoxy anthraquinone), polysassharides and one miscellaneous compound have been quantified as mark compounds for the quality control of *H. diffusa*. However, there were wide variations in the contents of these compounds, caused by samples from different sources and different collecting times (Table 3). Therefore, it is very urgent that a comprehensive method for ensuring the quality of *H. diffusa* be established.

## 5. Pharmacokinetics

The investigations about pharmacokinetics of *H. diffusa* are very scarce. After oral administration of *H. diffusa* in lipopolysaccharide-induced renal inflammation in mice, most compounds, including flavonoids, iridoid glycosides and anthraquinone, were found in plasma, and 12 compounds (eight flavonoids and four iridoid glycosides) were found in kidney, determined by UPLC-Q-TOF-MS/MS. The results indicated that flavonoids, iridoids and anthraquinones might be responsible for the protective effect of *H. diffusa* on renal inflammation [87]. Liu *et al.* [111] found that *p*-coumaric acid was a major metabolite of (*E*)-6-*O*-*p*-coumaroyl scandoside methyl ester in rat plasma after oral administration of a dose of 20 mg/kg. Compared with direct administration of *p*-coumaric acid, the absorption and elimination of *p*-coumaric acid were slower with administration of (*E*)-6-*O*-*p*-coumaroyl scandoside methyl ester. This was also confirmed by Yan *et al.* at 2011 [112]. Moreover, Ganbold *et al.* [62] investigated the bioavailability of *H. diffusa* by production of post-absorption samples using the Caco-2 cell model and confirmed that the decoction has good permeability (Papp = 3.575 × 10^−6^ cm/s) *in vitro* with no cytotoxic effect.

## 6. Conclusions

Although *H. diffusa* has been used in China for thousands of years as a heat-clearing and detoxifying medicine, it has become popular for its anti-cancer effect, especially in the Taiwan district. Modern research on *H. diffusa* has provided much evidence for its anti-cancer effect using *in vitro* and *in vivo* experiments and has tried to clarify the mechanism of its action. Meanwhile, its other activities, such as anti-oxidant, anti-inflammatory, anti-fibroblasts, immunomodulatory and neuroprotective effects, have been reported. The achievement of these therapeutic effects is due to the chemical composition of *H. diffusa*. One hundred and seventy-one compounds have been reported, including iridoids, flavonoids, anthraquinones, phenolic acids and their derivatives, sterols, triterpenes, polysaccharides, cyclotides, coumarins, alkaloids and volatile oils. Among these constituents, iridoids, flavonoids and anthraquinones are three main ingredients and may play an essential role in its activities. However, there is no official quality standard for the quality control of *H. diffusa*. The contents of bioactive compounds are significantly different in the samples from different sources and different collecting times. So, a feasible and reliable approach is urgently needed in considering the botanical origin and bioactive effects. Moreover, a relatively small number of pharmacokinetics studies have been summarized, and, therefore, it is difficult to evaluate the function of *H. diffusa* in the human body. Altogether, this review gives comprehensive information about *H. diffusa* and provides evidence for its clinical application and further development.

## Figures and Tables

**Table:** 

Compounds	R_1_	R_2_
45. Quercetin	OH	H
46. Rutin	OH	rutinose
47. Quercetin-3-*O*-β-d-glucopyranside	OH	β-d-Glc
48. Quercetin-3-*O*-β-d-galactopyranoside	OH	β-d-Gal
49. Quercetin-3-*O*-(2-*O*-glucopyranosyl)-β-d-glucopyranside	OH	β-d-Glc-(1→2)-d-Glc
50. Quercetin-3-*O*-(2-*O*-glucopyranosyl)-β-d-galactopyranoside	OH	β-d-Glc-(1→2)-d-Gal
51. Quercetin-3-*O*-sambubioside	OH	β-d-Xyl-(1→2)-d-Glc
52. Quercetin-3-*O*-[2-*O*-(6-*O*-*E*-ferloyl)-β-d-glucopyranosyl]-β-d-galactopyranoside	OH	6′-*O*-*E*-feruloyl-β-d-Glc-(1→2)-d-Gal
53. Quercetin-3-*O*-[2-*O*-(6-*O*-*E*-feruloyl)-β-d-glucopyranosyl]-β-d-glucopyanoside	OH	6′-*O*-*E*-feruloyl-β-d-Glc-(1→2)-d-Glc
54. Quercetin-3-*O*-[2-*O*-(6-*O*-*E*-sinapoyl)-β-d-glucopyranosyl]-β-d-glucopyanoside	OH	6′-*O*-*E*-sinapoyl-β-d-Glc-(1→2)-d-Glc
55. Quercetin-3-*O*-[2-*O*-(6-*O*-*E*-sinapoyl)-β-d-glucopyranosyl]-β-d-galactopyranoside	OH	6′-*O*-*E*-sinapoyl-β-d-Glc-(1→2)-d-Gal
56. Kaempferol	H	H
57. Kaempferol-3-*O*-β-d-glucopyranside	H	β-d-Glc
58. Kaempferol-3-*O*-β-d-galactopyranoside	H	β-d-Gal
59. Kaempferol-3-*O*-(2-*O*-β-d-glucopyranosyl)-β-d-galactopyranoside	H	β-d-Glc-(1→2)-d-Gal
60. Kaempferol-3-*O*-(6-*O*-α-l-rhamnosyl)-β-d-glucopyranside	H	α-l-Rha-(1→6)-β-d-Glc
61. Kaempferol-3-*O*-[2-*O*-(6-*O*-*E*-ferloyl)-β-d-glucopyranosyl]-β-d-glucopyranside	H	6′-*O*-*E*-feruloyl-β-d-Glc-(1→2)-d-Glc
62. Kaempferol-3-*O*-[2-*O*-(6-*O*-*E*-feruloyl)-β-d-glucopyranosyl]-β-d-galactopyranoside	H	6′-*O*-*E*-feruloyl-β-d-Glc-(1→2)-d-Gal

**Table:** 

Compounds	R_1_	R_2_	R_3_	R_4_	R_5_	R_6_
63. 2-Methyl-3-methoxy anthraquinone	H	CH_3_	OCH_3_	H	H	H
64. 2-Hydroxy-1,3-dimethoxy anthraquinone	OCH_3_	OH	OCH_3_	H	H	H
65. 2-Hydroxy-3-methyl-1-methoxy anthraquinone	OCH_3_	OH	CH_3_	H	H	H
66. 2-Hydroxy-3-methyl-4-methoxy anthraquinone	H	OH	CH_3_	OCH_3_	H	H
67. 2-Hydroxy-7-methyl-3-methoxy anthraquinone	H	OH	OCH_3_	H	H	CH_3_
68. 2-Hydroxy-1-methoxy-3-methyl anthraquinone	OCH_3_	OH	CH	H	H	H
69. 2-Hydroxy-3-methyl anthraquinone	H	OH	CH_3_	H	H	H
70. 2-Hydroxy-1-methoxy anthraquinone	OCH_3_	OH	H	H	H	H
71. 2-Hydroxy-4-methoxy anthraquinone	H	OH	H	OCH_3_	H	H
72. 2-Hydroxy-3-methoxy-7-methyl anthraquinone	H	OH	OCH_3_	H	H	CH_3_
73. 2-Hydroxy-6-methyl anthraquinone	H	OH	H	H	CH_3_	H
74. 2-Hydroxy-3-methoxy-6-methyl anthraquinone	H	OH	OCH_3_	H	CH_3_	H
75. 2,7-Dihydroxy-3-methyl anthraquinone	H	OH	CH_3_	H	H	OH
76. 3-Hydroxy-2-methyl anthraquinone	H	CH_3_	OH	H	H	H
77. 3-Hydroxy-2-methyl-4-methoxy anthraquinone	H	CH_3_	OH	OCH_3_	H	H
78. 2,3-Dimethoxy-6-methyl anthraquinone	H	OCH_3_	OCH_3_	H	CH_3_	H
79. 1,3-Dihydroxy-2-methyl anthraquinone	OH	CH_3_	OH	H	H	H
80. 1,7- Dihydroxy-6-methoxy-2-methyl anthraquinone	OH	CH_3_	H	H	OCH_3_	OH
81. 3-Hydroxy-2-methyl-4-methoxy anthraquinone	H	CH_3_	OH	OCH_3_	H	H
82. 2,6-Dihydroxy-3-methyl-4-methoxy anthraquinone	H	OH	CH_3_	OCH_3_	OH	H
83. 2,6-Dihydroxy-1-methoxy-3-methyl anthraquinone	OCH_3_	OH	CH_3_	H	OH	H
84. 1-Hydroxy-4-methoxy anthraquinone	OH	H	H	OCH_3_	H	H
85. 2-hydroxymethyl-1-hydroxy anthraquinone	OH	CH_2_OH	H	H	H	H
86. 2-hydroxymethyl anthraquinone	H	CH_2_OH	H	H	H	H

**Table 1:** Compounds of the *H. diffusa*.

NO.	Compound Name	Molecular Formula	Reference
**Iridoids**			
1	Asperuloside	C_18_H_22_O_11_	[13,14]
2	Deacetyl asperuloside	C_16_H_20_O_10_	[15]
3	Asperuloside acid	C_18_H_24_O_12_	[16]
4	Deacetyl asperulosidic acid	C_16_H_22_O_11_	[15]
5	Deacetyl asperulosidic acid methyl ester	C_17_H_24_O_11_	[15,17]
6	Geniposidic acid	C_16_H_22_O_10_	[18]
7	10-*O*-Acetyl geniposidic acid	C_18_H_24_O_11_	[15]
8	10-Dehydro geniposide	C_17_H_22_O_10_	[17]
9	10-Dehydro geniposidic acid	C_16_H_20_O_10_	[19]
10	Diffusoside A	C_1__9_H_2__8_O_1__1_	[20]
11	Diffusoside B	C_1__9_H_2__8_O_1__1_	[20]
12	Lupenylacetate	C_32_H_52_O_2_	[21]
13	Alpigenoside	C_18_H_28_O_12_	[15]
14	Oldenlandoside III	C_34_H_44_O_20_	[22]
15	5-*O*-Feruloyl scandoside methyl ester	C_27_H_32_O_14_	[23]
16	Hehycoryside C	C_23_H_26_O_11_	[22]
17	6-α-Hydro scandoside	C_16_H_22_O_11_	[24]
18	6-β-Hydro scandoside	C_16_H_22_O_11_	[24]
19	6-Dehydro scandoside	C_16_H_22_O_10_	[19]
20	6-α-Hydro scandoside methyl ester	C_1__7_H_2__4_O_11_	[24]
21	6-β-Hydro scandoside methyl ester	C_1__7_H_2__4_O_1__1_	[24]
22	6-α-Hydro-10-acetyl asperuloside acid	C_18_H_24_O_12_	[24]
23	6-β-Hydro-10-acetyl asperuloside acid	C_18_H_24_O_12_	[24]
24	6-*O*-Methoxyl cinnamoyl scandoside	C_2__7_H_32_O_1__3_	[23]
25	6-*O*-*p*-Hydro cinnamoyl scandoside	C_2__6_H_30_O_1__3_	[23]
26	(*E*)-6-*O*-*p*-Coumaroyl-10-*O*-formoxyl scandoside methyl ester	C_27_H_32_O_13_	[14]
27	(*E*)-6-*O*-*p*-Coumaroyl scandoside methyl ester	C_26_H_30_O_13_	[14,15,25]
28	(*Z*)-6-*O*-*p*-Coumaroyl scandoside methyl ester	C_26_H_30_O_13_	[18]
29	(*E*)-6-*O-p*-Methoxy cinnamoyl scandoside methyl ester	C_27_H_32_O_13_	[15,25,26]
30	(*Z*)-6-*O*-*p*-Methoxy cinnamoyl scandoside methyl ester	C_27_H_32_O_13_	[26]
31	(*E*)-6-*O*-Feruloyl scandoside methyl ester	C_27_H_32_O_14_	[15,25,26]
32	(*Z*)-6-*O*-Feruloyl scandoside methyl ester	C_27_H_32_O_14_	[27]
**Triterpenes**			
33	Arborinone	C_30_H_48_O	[28]
34	Isoarborinol	C_30_H_50_O	[28]
35	Oleanolic acid	C_30_H_48_O_3_	[19]
36	Ursolic acid	C_30_H_48_O_3_	[19]
**Flavonoids**			
37	Amentoflavone	C_30_H_18_O_10_	[26,29]
38	Chrysin-6-*C*-glucosyl-8-*C*-arabinosyl	C_26_H_2__8_O_13_	[22]
39	Chrysin-6-*C*-arabinosyl-8-*C*-glucosyl	C_26_H_2__8_O_13_	[22]
40	Oroxylin-A-*O*-glucuronic acid	C_22_H_20_O_11_	[22]
41	Wogonin-*O*-glucuronic acid	C_22_H_20_O_11_	[22]
42	5,7-Dihydroxy-3-methoxy flavonol	C_16_H_12_O_5_	[13]
43	5,7,4′-Trihydroxy flavonol	C_15_H_10_O_6_	[13]
44	5-Hydroxy-6,7,3′,4′-tetramethoxy flavone	C_19_H_18_O_7_	[21]
45	Quercetin	C_15_H_10_O_7_	[17,19,30]
46	Rutin	C_27_H_30_O_16_	[15,25,31]
47	Quercetin-3-*O*-β-d-glucopyranside	C_21_H_20_O_12_	[25,32,33]
48	Quercetin-3-*O*-β-d-galactopyranoside	C_21_H_20_O_1__2_	[32]
49	Quercetin-3-*O*-(2-*O*-glucopyranosyl)-β-d-glucopyranside	C_27_H_3__0_O_17_	[15,25,32,33]
50	Quercetin-3-*O*-(2-*O*-glucopyranosyl)-β-d-galactopyranoside	C_27_H_3__0_O_17_	[11,34]
51	Quercetin-3-*O*-sambubioside	C_26_H_28_O_16_	[15,25]
52	Quercetin-3-*O*-[2-*O*-(6-*O*-*E*-ferloyl)-β-d-glucopyranosyl]-β-d-galactopyranoside	C_37_H_38_O_20_	[11,34]
53	Quercetin-3-*O*-[2-*O*-(6-*O*-*E*-feruloyl)-β-d-glucopyranosyl]-β-d-glucopyanoside	C_37_H_38_O_20_	[11,15,25]
54	Quercetin-3-*O*-[2-*O*-(6-*O*-*E*-sinapoyl)-β-d-glucopyranosyl]-β-d-glucopyanoside	C_38_H_40_O_21_	[15]
55	Quercetin-3-*O*-[2-*O*-(6-*O*-*E*-sinapoyl)-β-d-glucopyranosyl]-β-d-galactopyranoside	C_38_H_40_O_21_	[25]
56	Kaempferol	C_15_H_10_O_6_	[17,35]
57	Kaempferol-3-*O*-β-d-glucopyranside	C_21_H_20_O_11_	[32]
58	Kaempferol-3-*O*-β-d-galactopyranoside	C_21_H_20_O_11_	[32]
59	Kaempferol-3-*O*-(2-*O*-β-d-glucopyranosyl)-β-d-galactopyranoside	C_27_H_30_O_16_	[11,25,34]
60	Kaempferol-3-*O*-(6-*O*-α-l-rhamnosyl)-β-d-glucopyranside	C_27_H_3__0_O_16_	[32]
61	Kaempferol-3-*O*-[2-O-(*E*-6-*O*-feruloyl)-β-d-glucopyranosyl]-β-d-glucopyranosyl	C_37_H_38_O_19_	[11,25,33]
62	Kaempferol-3-*O*-[2-*O*-(6-*O*-*E*-feruloyl)-β-d-glucopyranosyl]-β-d-galactopyranoside	C_37_H_38_O_19_	[21,34]
**Athraquinones**			
63	2-Methyl-3-methoxy anthraquinone	C_16_H_12_O_3_	[19]
64	2-Hydroxy-1,3-dimethoxy anthraquinone	C_16_H_1__2_O_5_	[29]
65	2-Hydroxy-3-methyl-1-methoxy anthraquinone	C_16_H_12_O_4_	[36]
66	2-Hydroxy-3-methyl-4-methoxy anthraquinone	C_16_H_12_O_4_	[37]
67	2-Hydroxy-7-methyl-3-methoxy anthraquinone	C_16_H_12_O_4_	[36]
68	2-Hydroxy-1-methoxy-3-methyl anthraquinone	C_16_H_14_O_4_	[31]
69	2-Hydroxy-3-methyl anthraquinone	C_15_H_10_O_3_	[17,27]
70	2-Hydroxy-1-methoxy anthraquinone	C_15_H_10_O_4_	[27,29]
71	2-Hydroxy-4-methoxy anthraquinone	C_15_H_10_O_4_	[38]
72	2-Hydroxy-3-methoxy-7-methyl anthraquinone	C_16_H_12_O_4_	[36]
73	2-Hydroxy-6-methyl anthraquinone	C_15_H_10_O_3_	[18]
74	2-Hydroxy-3-methoxy-6-methyl anthraquinone	C_16_H_12_O_4_	[18]
75	2,7-Dihydroxy-3-methyl anthraquinone	C_15_H_10_O_4_	[39]
76	3-Hydroxy-2-methyl anthraquinone	C_15_H_10_O_3_	[19]
77	3-Hydroxy-2-methyl-4-methoxy anthraquinone	C_16_H_12_O_4_	[40]
78	2,3-Dimethoxy-6-methyl anthraquinone	C_17_H_14_O_4_	[18]
79	1,3-Dihydroxy-2-methyl anthraquinone	C_15_H_10_O_4_	[41]
80	1,7- Dihydroxy-6-methoxy-2-methyl anthraquinone	C_16_H_12_O_5_	[41]
81	3-Hydroxy-2-methyl-4-methoxy anthraquinone	C_1__6_H_10_O_4_	[18]
82	2,6-Dihydroxy-3-methyl-4-methoxy anthraquinone	C_16_H_12_O_5_	[42]
83	2,6-Dihydroxy-1-methoxy-3-methyl anthraquinone	C_16_H_12_O_5_	[31]
84	1-Hydroxy-4-methoxy anthraquinone	C_15_H_10_O_4_	[43]
85	2-Hydroxymethy-1-hydroxy anthraquinone	C_15_H_10_O_4_	[5]
86	2-Hydroxymethyl anthraquinone	C_15_H_10_O_3_	[5]
**Phenolic acids and their derivatives**			
87	3,4-Dihydroxy benzoic acid	C_7_H_6_O_4_	[21]
88	4-Hydroxy-3-methoxy benzoic acid	C_8_H_8_O_4_	[30]
89	*trans*-Hydroxybenzoic acid	C_7_H_6_O_3_	[30]
90	4-Hydroxy-3,5-dimethoxy benzoic acid	C_9_H_10_O_5_	[30]
91	*p*-Coumaric acid	C_9_H_8_O_3_	[19,29]
92	*p*-Coumaric acid-*O*-glucopyranside	C_15_H_18_O_8_	[22]
93	Caffeic acid	C_9_H_8_O_4_	[21]
94	Caffeoyl hexoside	C_15_H_18_O_9_	[22]
95	Ferulic acid	C_10_H_10_O_4_	[41]
96	Ferulic acid hexoside	C_16_H_20_O_9_	[22]
97	p-Methoxy cinnamic acid	C_10_H_10_O_3_	[44]
98	4,4′-Dihydroxy-α-truxillic acid	C_18_H_16_O_6_	[44]
99	4,4′-Dimethoxyl-α-truxillic acid	C_19_H_18_O_6_	[45]
100	Octadecyl (*E*)-*p*-coumarate	C_27_H_44_O_3_	[46]
101	3-Caffeoyl quinic acid	C_16_H_18_O_9_	[22]
102	4-Caffeoyl quinic acid	C_16_H_18_O_9_	[22]
103	5-Caffeoyl quinic acid	C_16_H_18_O_9_	[22]
104	3-*р*-Coumaroyl quinic acid	C_16_H_18_O_8_	[22]
105	4-*р*-Coumaroyl quinic acid	C_16_H_18_O_8_	[22]
106	5-*р*-Coumaroyl quinic acid	C_16_H_18_O_8_	[22]
107	3-Feruloyl quinic acid	C_17_H_20_O_9_	[22]
108	4-Feruloyl quinic acid	C_17_H_20_O_9_	[22]
109	5-Feruloyl quinic acid	C_17_H_20_O_9_	[22]
**Sterols**			
110	Daucosterol	C_35_H_60_O_6_	[19]
111	β-Sitosterol	C_29_H_50_O	[19]
112	Stigmasterol	C_29_H_48_O	[17,19]
113	Stigmasterol-5,2-diene-3β, 7α-glycol	C_29_H_48_O_2_	[47]
**Volatile oils**			
114	6,10,14-Trimethyl-2-pentadecanone	C_18_H_36_O	[48]
115	Phytol	C_20_H_40_O	[48]
116	α-Cedrol	C_15_H_26_O	[48]
117	Tetradecanoic acid	C_14_H_28_O_2_	[48]
118	Hexadecanoic acid, methyl ester	C_17_H_34_O_2_	[48]
119	Hexadecanoic acid,	C_16_H_32_O_2_	[48]
121	1,2-Benzenediearboxylic acid isobutyl ester	C_16_H_22_O_4_	[48]
122	1,2-Benzenediearboxylic acid, bis(2-methylpropyl)ester	C_16_H_22_O_4_	[48]
123	9,12,15-Octadecatrienoic acid, methyl ester	C_19_H_32_O_2_	[48]
124	9-Octadecenoic acid	C_18_H_34_O_2_	[48]
125	9,12-Octadecenoic acid	C_18_H_32_O_2_	[48]
126	Ethyl linoleate	C_20_H_36_O_2_	[48]
127	Triethyl phosphate	C_6_H_15_O_4_P	[48]
128	4-Vinyl phenol	C_8_H_8_O	[48]
129	2-Methoxy-4-vinylphenol	C_9_H_10_O_2_	[48]
130	*n*-Pentadecanoic acid	C_15_H_30_O_2_	[48]
131	4,8,12,16-Tetramethyl heptadecan-4-olide	C_21_H_40_O_2_	[48]
132	2,6,10,14,18,22-Tetracosahexaene	C_30_H_50_	[48]
133	α-Terpineol	C_10_H_18_O	[11]
134	Geranyl acetate	C_12_H_20_O_2_	[11]
135	β-Ionone	C_13_H_20_O	[11]
136	Lauric acid	C_12_H_24_O_2_	[11]
137	Myristic acid	C_14_H_28_O_2_	[11]
138	Palmitic acid	C_16_H_32_O_2_	[11]
139	Linoleic acid	C_18_H_32_O_2_	[11]
140	β-Linalool	C_10_H_18_O	[11]
141	Isoborneol	C_10_H_18_O	[49]
142	3-(2-Propenyl)-cyclohexene	C_9_H_14_	[49]
143	2-Pentyl-furam	C_9_H_14_O	[49]
144	Cis-2-(2-pentenyl)-furan	C_9_H_12_O	[49]
145	Limonene	C_10_H_18_	[49]
146	3,7-Dimethyl-1,6-octadiem-3-ol	C_10_H_18_O	[49]
147	*trans*-5-Methyl-2-(1-methylethyl)-cyclohexanope	C_10_H_18_O	[49]
148	(1S-endo)-1,7,7-Trimethyl-bicyclo[2,2,1]heptan-2-ol	C_10_H_18_O	[49]
149	*p*-Menth-1-en-8-ol	C_10_H_18_O	[49]
150	Pulegone	C_10_H_16_O	[49]
151	4-(2,6,6-Trimethyl-1-cyclohexen-1-yl)-3-buten-2-one	C_13_H_20_O	[49]
152	Hexadecanal	C_16_H_32_O	[49]
153	2,6,10,14-Tetramethyl-hexadecane	C_20_H_42_	[49]
154	(*Z,Z*)-9,12-octadecadienoic acid	C_18_H_32_O_2_	[49]
155	(*Z*)-9,17-octadecadienal	C_18_H_32_O	[49]
156	Cis,cis,cis-7,10,13-hexadecatrienal	C_16_H_26_O	[49]
157	Oleic acid	C_18_H_34_O_2_	[49]
158	Hexaldehyde	C_6_H_12_O	[49]
159	Borneol	C_10_H_18_O	[49]
160	Docosane	C_22_H_46_	[49]
161	Tetracosane	C_24_H_50_	[49]
162	Hexacosane	C_26_H_54_	[49]
163	Heptacosane	C_27_H_56_	[49]
**Polysaccharides**			
164	ODP-1		[50]
**Cyclotides**			
165	CD1		[51]
166	CD2		[51]
167	CD3		[51]
**Coumarins**			
168	7-Hydroxy-6-methoxy-Coumarin	C_10_H_8_O_4_	[17]
169	Esculetin	C_9_H_6_O_4_	[46]
**Alkaloids**			
170	10(*S*)-hydroxy pheophytin a	C_55_H_74_N_4_O_6_	[52]
171	Aurantiamide acetate	C_27_H_28_N_2_O_4_	[46]

**Table 2:** Pharmacological effects of *H. diffusa*.

Activities	Model	Formulation/Dosage/Extract		Reference
**Anti-tumor activity**				
Colorectal cancer	HT-29 cells	Ethanol extract	The extract suppressed HT-29 cell growth and induced apoptosis via inactivation of the IL-6/STAT3-signaling pathway.	[2]
	HT-29 cells	Ethanol extract	The extract reduced HT-29 cell viability and survival. It could suppress cancer cell proliferation by blocking the cell cycle, preventing G_1_ to S progression, and reducing mRNA expression of pro-proliferative PCNA, Cyclin D1 and CDK4, but increasing that of anti-proliferative p21.	[55]
	HT-29 cells	Ethanol extract	The extract induced the HT-29 cell morphological changes and reduced cell viability. In addition, the extract treatment resulted in DNA fragmentation, loss of plasma membrane asymmetry, collapse of mitochondrial membrane potential, activation of caspase-9 and caspase-3 and increase of the ratio of pro-apoptotic Bax to anti-apoptotic Bcl-2.	[56]
	HT-29 cells	Ethanol extract	The extract treatment downregulated the mRNA and protein expression levels of VEGF-A in HT-29 human colon carcinoma cells.	[57]
	HT-29 cells	Ethanol extract	The extract inhibits colorectal cancer growth *in vivo* via inhibition of SHH-mediated tumor angiogenesis.	[58]
	CRC mouse xenograft model	Ethanol extract	The extract inhibited the expression of the gene VEGF-A and VEGFR2, thus, suppressed the activation of Sonic hedgehog (SHH)-signaling in CRC xenograft tumors; it inhibits colorectal cancer growth.	[58]
	CRC mouse xenograft model	Ethanol extract	The extract suppressed the STAT3 pathway by suppressing STAT3 phosphorylation in tumor tissues, altering the expression pattern of target genes of Cyclin D1, CDK4 and Bcl-2, as well as upregulating p21 and Bax.	[59]
	CT-26 cells	Ethanol extract	The extract can inhibit the proliferation of CT-26 colon cancer cells from BALB/c mice in a time- and dose- dependent manner.	[60]
	HCT-8/5-FU cells	Ethanol extracts	The extract treatment significantly reduced the cell viability of HCT-8/5-FU cells by downregulating the expression of P-gp and ABCG2.	[61]
	Caco-2 cells	Aqueous extracts	The decoction of *H. diffusa* and its fraction 9 contained sufficient ursolic acid and oleanolic acid to possibly induce apoptosis of Caco-2 cells.	[62]
	Caco-2 cells	Nine pure compounds isolated from *H. diffusa*	2-Hydroxymethy-1-hydroxy anthraquinone (IC_50_ 45 mM) and ursolic acid (IC_50_ 71 mM) exhibited the highest inhibition of Caco-2 cell proliferation.	[5]
Leukemia	CEM cells	Aqueous extract	The extract inhibited Leukemia CEM cells growth in time- and concentration-dependent manners. And the inhibition mechanism has greater correlation with the upregulation of P53 expression.	[63]
	BALB/c mice	Aqueous extract	The extract had anti-leukemia effects on WEHI-3 cell-induced leukemia *in vivo*.	[64]
	HL-60 cells	*H. diffusa* injection	The extract could induce HL-60 cells differentiation, and suppress the expression of the anti-apoptosis-related gene to inhibit the growth of HL-60 cells.	[65]
	HL-60 cells, WEHI-3 cells	Ethanol extract	The extract inhibited the cell proliferation of HL-60 cells. It triggered an arrest of HL-60 cells at the G_0_/G_1_ phase and sub-G_1_ population, provoked DNA condensation and DNA damage, but the activities of caspase-3, caspase-8, and caspase-9 were elevated in *H. diffusa*-treated HL-60 cells.	[66]
	U937 cells	2-Hydroxy-3-methyl anthraquinone	2-Hydroxy-3-methyl anthraquinone enhanced apoptosis of U937 cells through the activation of p-p38MAPK and downregulation of p-ERK1/2.	[67]
	THP-1 Cells	2-Hydroxy-3-methyl anthraquinone	2-Hydroxy-3-methyl anthraquinone induced THP-1 cell apoptosis, which was associated with a more prominent induction expression of Fas/FasL, DR4 and TRAIL. Moreover, 2-Hydroxy-3-methylanthraquinone treatment resulted in activation of caspase-8.	[68]
Liver cancer	H22 mice	Aqueous extract	The extract had an inhibitory effect on the metastasis of hepatocarcinoma in blood.	[69]
	HepG2 cells	Aqueous extract	The extract remarkably inhibited HepG2 cell proliferation via arrest of HepG2 cells at the G_0_/G_1_ phase and induction of S phase delay. In addition, the extract potentiated the anticancer effect of low-dose 5-FU in the absence of overt toxicity by downregulating the mRNA and protein levels of CDK2, cyclin E and E2F1.	[70]
	MHCC97-H cells	Total flavones extract	The extract treatment reduced the level of E-cadherin protein and increased the expression of vimentin protein in TGF-β1-induced MHCC97-H.	[71]
	HepG2 cells	1,3-Dihydroxy-2-Methylanthraquinone Ethyl acetate extract	Both 1,3-Dihydroxy-2-Methylanthraquinone and ethyl acetate extract exhibited an inhibitory effect on HepG2 cells, resulting in in upregulation of Bax, p53, Fas, FasL, p21 and cytoplasmic cytochrome C levels and caspase-3, -8, -9 proteases activities, while downregulating Bcl-2, mitochondrial cytochrome C, cyclin E and CDK 2 in a dose-dependent manner.	[72]
	HepG2 cells	Nine pure compounds isolated from *H. diffusa*	Ursolic acid exhibited a strong inhibition of cell survival with C_50_ 37 mM.	[5]
	HepG2 cells	2-Hydroxy-3-methyl anthraquinone 1-Methoxy-2-hydroxy anthraquinone	Both compounds showed inhibitory activity against protein tyrosine kinases v-src and pp60src and arrested the growth of HepG2 cancer cells.	[38]
Lung cancer	A549 cells, H1355 cells, LLC cells	Ethanol extract	The extract suppressed the cell proliferation of A549 and H1355 cells as well as reduced cell viability in a concentration-dependent manner.	[66]
	SPC-1-A cells	2-Hydroxy-3-methyl anthraquinone 1-Methoxy-2-hydroxy anthraquinone	Both compounds showed inhibitory activity against protein tyrosine kinases v-src and pp60src and arrested the growth of SPC-1-A.	[38]
Breast cancer	MCF-7 cells	Compounds of anthraquinones, iridoid glucosides, stigmasterols and alkaloids/flavonoids	Alkaloids/flavonoids possessed antitumor activity against the human breast cancer cell line MCF7	[73]
	MCF-7 cells	Methyl anthraquinone	Methyl anthraquinone-induced MCF-7 cells apoptosis via Ca^2+^/calpain/caspase-4 pathway.	[74]
	Bcap37 cells	2-Hydroxy-3-methyl anthraquinone, 1-Methoxy-2-hydroxy anthraquinone	Both compounds showed inhibitory activity against protein tyrosine kinases v-src and pp60src and arrested the growth of Bcap37 cells.	[38]
Cervical tumor	Nude mouse model	Aqueous extract	The extract had an inhibitory effect on cervical cancer cells with the expression of Ki-67 protein significantly decreased, and the mean survival time of the mice was significantly extended.	[3]
	HeLa cells	Nine pure compounds isolated from *H. diffusa*	2-Hydroxymethy-1-hydroxy anthraquinone exhibited the strongest inhibitory effect on cell viability.	[5]
Prostate Cancer	DU145 cells, PC-3 cells LNCaP cells	Nine pure compounds isolated from *H. diffusa*	2-Methyl-3-methoxy anthraquinone, 2-hydroxy-3-methyl anthraquinone and ursolic acid exhibited inhibitory effects on prostate cancer cell survival.	[5]
	PC3 cells LNCaP cells	6-*O*-(*E*)-*p*-Coumaroyl scandoside methyl ester 10(*S*)-Hydroxy pheophytin	Two compounds showed a moderate anti-proliferation effect on PC3 human androgen-independent prostate cancer cells, while 10(S)-hydroxy pheophytin also showed a strong anti-proliferation effect on LNCaP human androgen-sensitive prostate cancer cells.	[52]
Multiple myeloma	RPMI 8226 cells	Nine pure compounds isolated from *H. diffusa*	2-Hydroxymethy-1-hydroxy anthraquinone exhibited the strongest inhibition of RPMI 8226cells growth.	[5]
	RPMI 8226 cells	Polysaccharides extracts	Polysaccharides extracts suppressed the growth of RPMI 8226 cells in a dose- and time-dependent manner.	[75]
	RPMI 8226 cells	*H*. *diffusa* injection	*H*. *diffusa* injection could inhibit the proliferation of RPMI 8226 cells.	[76]
Others	B16F10 cells	Ethanol extract	The extract suppressed the cell proliferation of B16F10 cells as well as reducing cell viability in a concentration-dependent manner.	[66]
	S180 cells	Decoction, lipophilic extract, crude polysaccharide	Lipophilic extract and crude polysaccharide showed anti-tumor activities and a protective effect on chemotherapeutic damage. However, the aqueous extract had no marked anti-tumor effect on S-180 cells.	[77]
	MG-63cells	*H*. *diffusa* injection	*H*. *diffusa* injection could inhibit the proliferation of MG-63 cells, and Bax gene expression was significantly increased.	[78]
	MG-63 cells	*H*. *diffusa* injection	*H*. *diffusa* injection could induce the apoptosis of MG-63 cells by increasing Bax gene expression in a concentration-dependent manner.	[79]
	MG-63 cells	Aqueous extract	*H*. *diffusa,* combined with cisplatin, had a stronger inhibitory effect than the single agents in MG-63 cells with IC_50_164.6 and 5.0 μL/mL, respectively. As a result, *H*. *diffusa* could alter anti-apoptotic (Bax and Bad) and pro-apoptotic protein (Bcl-xl and Bcl-2) expression, and it elevated the levels of caspase-3 and caspase-8.	[80]
	U87 cells	Aqueous extract	The extract suppressed U87 cells growth in a dose- and time-dependent manner.	[4]
Angiogenesis	1.Breast tumor-bearing BALB/c mice 2. Zebrafish embryo model 3. Human endothelial cells 4. C57BL/6 mice	4-Vinyl phenol	4-Vinyl phenol was demonstrated with anti-angiogenic activity *in vitro* and *in vivo*.	[81]
**Immunomodulatory effect**				
	Normal BALB/c mice	Ethanol Extract	The extract has promoted immune responses in normal BALB/c mice.	[82]
	Immunosuppression mice induced by cyclophosphamide	Polysaccharides extracts	The extract could improve the clearance index, phagocytic index, and the index of the thymus and spleen of immunosuppression mice.	[50]
	Inmmunosuppressed mice induced by cyclophosphamide	Total flavonoids extract	The extract enhanced specific and non-specific immunity.	[83]
**Antioxidant effects**				
		The extract from methanol, acetone and 80% alcohol	The extraction with 80% alcohol has the strongest antioxidant activity on DPPH assay.	[84]
		The extract from water, ethanol, acetone, chloroform, ether, petroleum benzine	Acetone extract had the strongest antioxidant effect.	[85]
	LO_2_ cells	Aqueous extract	The aqueous extract exerted a good antioxidant effect in DPPH assay with a 50% scavenging concentration at 0.153 mg/mL. Aqueous extract treatment reversed H_2_O_2_-induced activation of the MEK/ERK pathway and H_2_O_2_-induced inhibition of the P13-K/AKT/GSK3b pathway in LO_2_ cells. This may be due to the improvement activity of the aqueous extract of H. diffusa on the antioxidant defense system.	[86]
		Twelve pure compounds isolated from *H. diffusa*	All compounds showed antioxidant effects on xanthine oxidase inhibition, xanthine-xanthine oxidase cytochrome c and TBA-MDA systems.	[33]
**Anti-inflammatory effect**				
	Lipopolysaccharide-induced renal inflammation mice	Aqueous extract	The extract protected renal tissues, significantly suppressed the production of TNF-α, IL-1, IL-6 and MCP-1, as well as significantly promoted the production of IL-10 in serum and renal tissues.	[87]
	RAW 264.7 cells	Total flavonoids extract	The extract treatment on LPS-stimulated RAW 264.7 cells, reduced expression of iNOS, TNF-α, IL-6 and IL-1β, as well as suppressing phosphorylation of IκB p38, JNK and ERK1/2 in a concentration-dependent manner, indicating that the anti-inflammatory activity of total flavonoids had a close relationship with the NF-κB and MAPK signaling pathways.	[88]
**Neuroprotective effect**				
	Rat cortical cells damaged by l-glutamate	Methanolic extract, five flavonoids and four *O*-acylated iridoid glycosides	All compounds exhibited significant neuroprotective activity in primary cultures of rat cortical cells damaged by l-glutamate.	[34]
**Anti-fibrosis effect**				
	Ras oncogene-transformed R6 cells	Oleanolic acid	Oleanolic acid inhibits the growth of ras oncogene-transformed R6 cells. Oleanolic acid-mediated growth inhibition of transformed cells does not require direct cell–cell contact between normal and ras-transformed cells.	[89]

**Table 3:** Quantitative analysis for the quality control of *H. diffusa*.

Analytes	Method	Results	Reference
Deacetyl asperulosidic acid methyl ester	HPLC	The contents of deacetyl asperulosidic acid methyl ester of 22 batches were from 0.31 to 3.34 mg/g.	[93]
Oleanolic acid	TLC	The contents of oleanolic acid of 3 batches were from 1.63% to 1.72%	[94]
Isoscutellarein	HPLC	The contents of isoscutellarein have a close relationship with the collecting times and were also different in leaves (1.11–2.72 mg/g) and stem (0.35–0.94 mg/g).	[95]
*p*-Coumaric acid	HPLC	The contents of *p*-coumaric acid in the injection of *H. diffusa* from four manufacturers ranged from 0.34 to 0.49 mg/mL.	[96]
*p*-Coumaric acid	HPLC	The contents of *p*-coumaric acid of 13 batches were from 0.46 to1.88 mg/mL	[97]
3,4-Dihydroxy methyl benzoate	HPLC	The contents of 3,4-dihydroxy methyl benzoate of 8 batches were from 40.8 to 87.0 μg/g.	[98]
Polysassharides	UV	Polysassharides have been determined by the phenol-sulfuric acid method by spectrophosured at 490 nm, and the content was 15.10%.	[99]
Ursolic acid Oleanolic acid	HPLC	Six batches have been determined with the contents of 1.75–3.37 mg/g for ursolic acid and 0.50–0.80 mg/g for oleanolic acid, indicating that the ursolic acid and oleanolic acid content in the samples from different sources were significantly different.	[100]
Ursolic acid Oleanolic acid	HPLC	The contents of ursolic acid and oleanolic acid have a close relationship with the collecting time. The range of contents was 1.17–3.75 and 0.19–0.96 mg/g for ursolic acid and oleanolic acid, respectively.	[101]
Ursolic acid Oleanolic acid	HPLC	The contents of ursolic acid and oleanolic acid were 0.51%–0.58% and 0.11%–0.14%, respectively. And the contents of the whole herb were slightly lower than those of the overground part for both of the two compounds.	[102]
Ursolic acid Oleanolic acid	HPLC-MS/MS	The contents of ursolic acid and oleanolic acid for 10 batches were 0.15%–0.65% and 0.06%–0.17%, respectively.	[103]
2-Hydroxy-3-methoxy-7-methyl anthraquinone 2-Hydroxy-1-methoxy anthraquinone	HPLC	The contents were 0.16–0.51 and 0.22–0.49 mg/g for 2-hydroxy-3-methoxy-7-methyl anthraquinone and 2-hydroxy-1-methoxyanthraquinone, respectively.	[104]
Asperuloside E-6-*O*-*p*-Coumaroyl scandoside methyl ester E-6-*O*-*p*-Coumaroyl scandoside methyl ester-10-methyl ether	HPLC	The contents of asperuloside, E-6-*O*-*p*-coumaroyl scandoside methyl ester and E-6-*O*-*p*-coumaroyl scandoside methyl ester-10-methyl ether have been determined in twenty-three batches. The result was that the contents of the compounds were significantly varied among the different samples. The concentration ranges were 0–7.885, 1.104–7.159 and 0–1.795 mg/g for asperuloside, E-6-*O*-*p*-coumaroyl scandoside methyl ester and E-6-*O*-*p*-coumaroyl scandoside methyl ester-10-methyl ether, respectively.	[105]
3,4-Dihydroxy methyl benzoate *p*-Coumaric acid Ferulic acid (*E*)-6-*O*-*p*-Coumaroyl scandoside methyl ester	HPLC	Four compounds have been quantified in the injection of *H. diffusa* with contents of 2.25–2.63, 7.02–7.15, 0.96–1.17 and 7.16–7.33 g/L for 3,4-dihydroxy methyl benzoate, *p*-coumaric acid, ferulic acid and (*E*)-6-*O*-*p*-coumaroyl scandoside methyl ester, respectively.	[106]
Geniposidic acid Ursolic acid Quercetin *p*-Coumaric acid	CE	Four compounds have been quantified in the injection of *H. diffusa* with contents of 1.004, 1.182, 0.110 and 0.067 mg/g for ursolic acid, geniposidic acid, quercetin and *p*-coumaric acid, respectively.	[107]
Asperuloside acid Asperuloside (*E*)-6-*O*-Feruloyl scandoside methyl ester (*E*)-6-*O*-*p*-Coumaroyl scandoside methyl ester Scandoside methyl ester	HPLC	The contents were 1.57–5.93, 1.45–3.86, 1.82–3.23, 1.54–3.82 and 1.49–4.11 mg/g for asperuloside acid, asperuloside, (*E*)-6-*O*-feruloyl scandoside methyl ester, (*E*)-6-*O*-*p*-coumaroyl scandoside methyl ester and scandoside methyl ester, respectively, and they were very different in different batches.	[108]
Quercetin-3-*O*-sambubioside Quercetin-3-*O*-β-d-glucopyranside Kaempferol-3-*O*-β-d-glucopyranside Rutin Quercetin Kaempferol	HPLC	Six compounds from eight batches of *H. diffusa* have been quantified with contents of 1.36–6.32, 0.98–10.23, 0.79–7.98, 4.92–15.78, 0.52–1.72 and 0.75–2.15 mg/g for quercetin-3-*O*-sambubioside, quercetin-3-*O*-β-d-glucopyranside, kaempferol-3-*O*-β-d-glucopyranside, rutin, quercetin and kaempferol, respectively, indicating that the contents for these compounds were quite different from different regions.	[109]
Desacetyl asperulosidic acid Asperuloside Aesculetin Coumaric acid Ferulic acid Quercetin Kaempferol	HPLC	Seven compounds from six batches of *H. diffusa* have been quantified with contents of 42.48 ± 1.43, 63.76 ± 1.01, 1765 ± 0.69, 881.9 ± 0.74, 86.99 ± 1.65, 1395 ± 0.731 and 902.2 ± 0.82 μg/g for desacetyl asperulosidic acid, asperuloside, aesculetin, coumaric acid, ferulic acid, quercetin, kaempferol, respectively.	[110]
